# Comparison of the DNBSEQ platform and Illumina HiSeq 2000 for bacterial genome assembly

**DOI:** 10.1038/s41598-024-51725-0

**Published:** 2024-01-14

**Authors:** Tongyuan Hu, Jianwei Chen, Xiaoqian Lin, Wenxin He, Hewei Liang, Mengmeng Wang, Wenxi Li, Zhinan Wu, Mo Han, Xin Jin, Karsten Kristiansen, Liang Xiao, Yuanqiang Zou

**Affiliations:** 1 BGI Research, Shenzhen, 518083 China; 2 BGI Research, Wuhan, 430074 China; 3 School of Bioscience and Biotechnology, South China University of Technology, Guangzhou, 510006 China; 4 Laboratory of Genomics and Molecular Biomedicine, Department of Biology, University of Copenhagen, Universitetsparken 13, 2100 Copenhagen, Denmark; 5 Shenzhen Engineering Laboratory of Detection and Intervention of Human Intestinal Microbiome, BGI Research, Shenzhen, 518083 China

**Keywords:** Biological techniques, Biotechnology, Computational biology and bioinformatics, Genetics, Microbiology, Bacteria, Microbial communities, Environmental microbiology, Microbial genetics

## Abstract

The Illumina HiSeq platform has been a commonly used option for bacterial genome sequencing. Now the BGI DNA nanoball (DNB) nanoarrays platform may provide an alternative platform for sequencing of bacterial genomes. To explore the impact of sequencing platforms on bacterial genome assembly, quality assessment, sequence alignment, functional annotation, mutation detection, and metagenome mapping, we compared genome assemblies based on sequencing of cultured bacterial species using the HiSeq 2000 and BGISEQ-500 platforms. In addition, simulated reads were used to evaluate the impact of insert size on genome assembly. Genome assemblies based on BGISEQ-500 sequencing exhibited higher completeness and fewer N bases in high GC genomes, whereas HiSeq 2000 assemblies exhibited higher N50. The majority of assembly assessment parameters, sequences of 16S rRNA genes and genomes, numbers of single nucleotide variants (SNV), and mapping to metagenome data did not differ significantly between platforms. More insertions were detected in HiSeq 2000 genome assemblies, whereas more deletions were detected in BGISEQ-500 genome assemblies. Insert size had no significant impact on genome assembly. Taken together, our results suggest that DNBSEQ platforms would be a valid substitute for HiSeq 2000 for bacterial genome sequencing.

## Introduction

Metagenomics has provided important information on the composition and functional potentials of the gut microbiota and associations between gut bacteria and complex phenotypic traits^1,2^. However, in part due to limited availability of cultivated bacterial strains and regulatory issues, causal relations have been difficult to establish in relation to human health and disease^3^. Consequently, cultivation and bacterial genome sequencing have attracted increased attention to provide updated taxonomic annotation and expanded resources of cultivated bacterial isolates and genome references^4–6^.

Illumina HiSeq/MiSeq, Roche-454, and Ion Torrent Personal Genome Machine (PGM) have been adopted for bacterial genome sequencing and metagenomic research for many years, with the Illumina HiSeq platform being a widely used sequencing platform owing to its ability to provide rapid and accurate analysis of entire bacterial genomes. BGISEQ-500 and later developed versions, employing combinatorial probe-anchor^7^, synthesis (cPAS)-based sequencing combined with DNB nanoarrays have contributed significantly to advance DNA and RNA sequencing of humans^8^, animals^9,10^, and plants^11,12^. Compared to Illumina sequencers, DNBSEQ sequencers have produced reads of at least similar quality in studies of genomes^13–15^, exomes^16,17^, transcriptomes^12,18^, and metagenomes^19^.

In a recent benchmarking study, the DNBSEQ platform was reported to provide the lowest sequencing error rates among short-read technologies^8^. Thus, the BGISEQ-500 sequencer and updated versions have the potential to be a perfect substitute for Illumina platforms to satisfy the increasing demands for cultivated bacterial genome sequencing. Here we performed a comparison on bacterial genome assembly using sequencing data of BGISEQ-500 and Illumina HiSeq 2000 in relation to genome quality assessment, genome alignment, functional annotation, mutation detection, and metagenome mapping. Considering the potential contamination in sequencing and potential insert size bias in the DNB technology^20^, we simulated sequencing reads and analyzed the impact of sequence contamination and insert size on genome assembly.

## Results

### Strains collection and taxonomic information

In this study, we included 76 bacterial strains, comprising 64 unique species from the project of the Culturable Genome Reference version two (CGR2)^4,21^ deposited in China National GeneBank (CNGB) with accession numbers CNP0000126 and CNP0001833. These strains were sequenced on both BGISEQ-500 and Illumina HiSeq 2000 to yield 152 shotgun sequencing datasets. Through genome assembly and taxonomic annotation, these strains could be classified into 5 phyla (Firmicutes 32 strains, Bacteroidota 26 strains, Actinobacteriota 10 strains, Proteobacteria 7 strains, Fusobacteriota 1 strain), 34 genera, and 64 species (Supplementary Table S1). These representative bacteria, which cover the main phyla of the human gut microbiota were selected for the comparison of the two sequencing platforms.

### Quality assessment of genome assemblies

**Figure 1:**
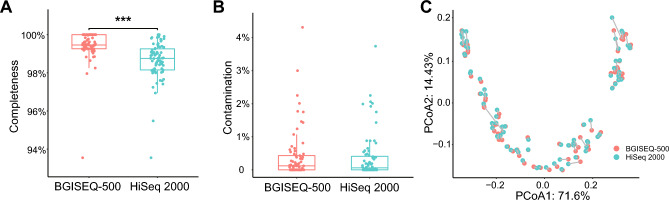
Quality assessment of genome assemblies. (**A**) Completeness and (**B**) contamination of genome assemblies generated from BGISEQ-500 sequencing data and HiSeq 2000 sequencing data. (**C**) PCoA of all assembly parameters based on Jaccard dissimilarity. Red: BGISEQ-500, Blue: HiSeq 2000.

All the 152 genome assemblies from both BGISEQ-500 and HiSeq 2000 were high-quality with completeness higher than 93% and contamination of less than 5% (Supplementary Table S2). Wilcoxon tests showed that the completeness of genome assemblies from BGISEQ-500 was significantly higher than that from HiSeq 2000 (p < 0.001) (Fig. 1A) and similar results were also shown for assemblies of GC percentage higher than 40% and less than 60% (Supplementary Fig. S1A,B). There was no significant difference in the contamination between assemblies using data from BGISEQ-500 and HiSeq 2000 (Fig. 1B).

We assessed these assemblies by paired comparison of the output of QUAST (Supplementary Table S2). The comparison of the mean values of assembly parameters showed that the numbers of contigs and numbers of N per 100Kb were lower, and the length of the largest contig and N50 were higher in HiSeq 2000 assemblies compared to BGISEQ-500 assemblies (Supplementary Fig. S2A–D). However, the number of N per 100Kb was lower in BGISEQ-500 assemblies (GC content > 60%). The length of genomes based on data from the two platforms was extremely consistent (Supplementary Fig. S2E). To evaluate all the assembly parameters from QUAST, PCoA (Principal Coordinates Analysis) with Jaccard dissimilarity was used and the results showed that the assemblies from the same strain were close together, irrespective of the platform (Fig. 1C).

### Sequence similarity of 16S rDNA, whole genome, and mutation detection

**Figure 2:**
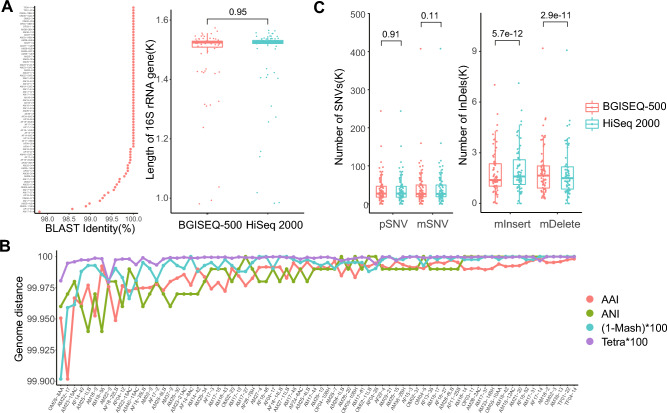
Sequence similarity of 16S rRNA genes and whole genomes, and mutation detection. (**A**) Sequence alignment and length of 16S rRNA genes. (**B**) Genome distance analysis using the distance algorithms AAI, ANI, Mash, and Tetra. (**C**) Comparison of numbers of SNVs and InDels. pSNV: SNVs called by Parsnp, mSNV: SNVs called by MUMmer, mInsert, and mDelete: Inserts and Deletions detected by MUMmer.

The 16S rRNA gene is the most commonly used marker in bacterial taxonomy analysis. BLAST alignment (Fig. 2A) showed that 16S rDNA predicted from paired genomes possessed similar sequences, with 72 paired sequence identity being higher than 99%. There was no difference in the length of the 16S rDNA sequences of 76 paired genome assemblies (Fig. 2A).

AAI (average amino acid identity), ANI (average nucleotide identity), Tetra (Tetra-nucleotide signature) correlation^22^, and Mash distance have often been used in establishing clusters of species at the genome level. These genome dissimilarity parameters were calculated to compare the differences between the pairwise genome assemblies from the two platforms. All pairwise ANIs and (1 − MASH)*100 were higher than 99.9, AAIs were higher than 99.935, and Tetras were above 99.975 (Fig. 2B). ANI > 95%, Tetra > 0.99, AAI > 95%, and MASH < 0.05 were used to evaluate whether two genomes should be considered as members of the same genomic species. The comparisons supported that the pairwise genomes from the two platforms were extremely close and did not differ significantly.

Seventy-one genomes were downloaded from the NCBI genome database as references (Supplementary Table S3). Parsnp and MUMmer were used as the main programs to align genome assemblies of BGISEQ-500 or HiSeq 2000 data to reference genomes, SNV and InDel were subsequently extracted from alignments. The numbers of SNV called by MUMmer were higher than those called using Parsnp. The platforms had no significant effect on SNV calling (Fig. 2C). Compared to SNV, more insertions were detected in HiSeq 2000 genome assemblies (p = 5.6e−12) and more deletions were detected in BGISEQ-500 genome assemblies (p = 2.9e−11) (Fig. 2C).

### Genome collinearity and functional regions assessment

**Figure 3:**
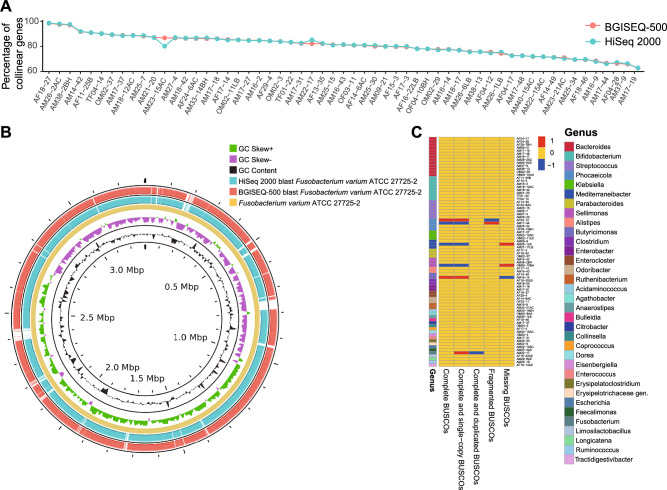
Genome collinearity and functional regions assessment. (**A**) Collinearity between genome assemblies and reference genomes. (**B**) Graphical circular map generated from genome assemblies of AM22-17 and reference genome *Fusobacterium varium* ATCC 27725-2. (**C**) Comparison of numbers of genes by the BUSCO assessment tool.

To conduct genomic collinearity analysis, genome assemblies of BGISEQ-500 and HiSeq 2000 were mapped to reference genomes. The result showed the percentage of collinear genes in the mapping of BGISEQ-500 assemblies was significantly correlated with that in the mapping of HiSeq 2000 assemblies (Pearson coefficient 0.992, p < 0.001) (Fig. 3A, and Supplementary Table S4). Although the AAI of AM22-17 assemblies from BGISEQ-500 and HiSeq 2000 was lower than that of other pairs, they had a high degree of genome collinearity with 5168 collinear genes (85.35%) (Fig. 3B). The result of prokaryotic genome annotation by Prokka showed that almost all paired genome assemblies (74/76) had the same numbers of functional regions, including the numbers of enzymes, COGs (Cluster of Orthologous Groups), genes, CDSs (coding sequences), tRNAs (transfer RNAs), rRNAs (ribosomal RNAs) and tmRNAs (transfer-messenger RNAs) (Supplementary Table S5). Genome assembly and annotation completeness were also evaluated by BUSCO (Benchmarking Universal Single-Copy Orthologues). Comparisons of the numbers of BUSCOs showed that only one difference occurred in five complete BUSCOs, six complete and single-copy BUSCOs, one complete and duplicated BUSCOs, two fragmented BUSCOs, and three missing BUSCOs in the 76 paired genome assemblies (Fig. 3C, and Supplementary Table S6).

### Distribution of genome assemblies in metagenome cohort

**Figure 4:**
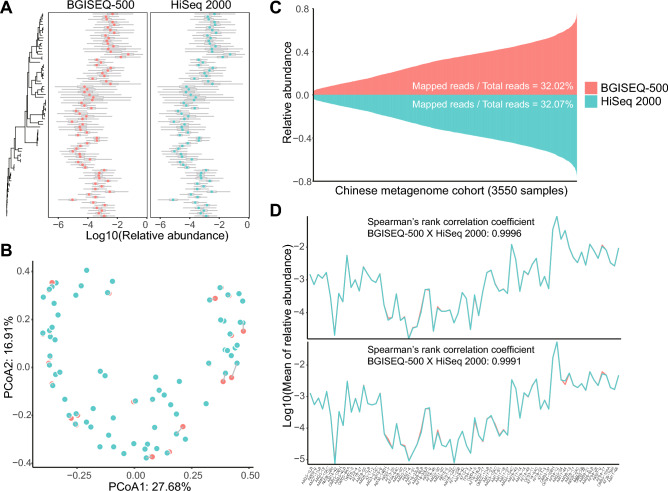
Distribution of genome assemblies in the metagenome cohort. (**A**) Relative abundance of paired genome assemblies in the metagenome cohort. (**B**) PCoA of platform effect on genome assembly with metagenomic reads mapping. (**C**) The sum of relative abundances of genome assemblies in each metagenome sample. (**D**) Mean and median of relative abundance of each genome assembly in the metagenome cohort. Red: BGISEQ-500, Blue: HiSeq 2000.

To identify the impact of sequencing platform on metagenomic reads mapping, the distribution of genome assemblies from BGISEQ-500 and HiSeq 2000 in a Chinese healthy cohort was analyzed (Fig. 4A). Beta-diversity showed that there was no difference between genome assemblies from BGISEQ-500 and HiSeq 2000 (p = 0.99) (Fig. 4B). The relative abundance of BGISEQ-500 assemblies and HiSeq 2000 assemblies in metagenomes were very similar; for both the sums of relative abundance were about 32% (Fig. 4C). In addition, the means and medians of the relative abundance of genome assemblies from the two platforms had a significant correlation, with coefficient of greater than 0.99 (Fig. 4D). These results demonstrate that the use of the two platforms for bacteria genome sequencing has no significant impact on sequence mapping in metagenomic data analysis.

### The impact of sequence contamination and insert size on genome assembly

**Figure 5:**
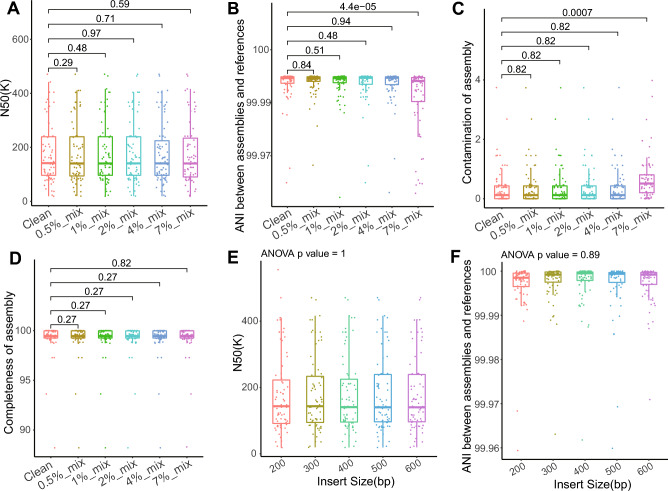
The impact of sequence contamination and insert size on assembly. (**A**) N50 of genome assemblies generated from simulated reads mixed with 0–7% contamination. (**B**) ANI between references and genome assemblies generated from simulated reads mixed with 0–7% contamination. (**C**,**D**) Contamination and completeness of genome assemblies generated from simulated reads mixed with 0–7% contamination. (**E**) N50 of genome assemblies generated from simulated reads with insert sizes 200–600 bp. (**F**) ANI between genome assemblies generated from simulated reads with insert sizes 200–600 bp.

Three million reads were simulated for each reference genome with a percentage of contamination reads from 0 to 7%. Compared to clean genomes, only genomes mixed with 7% contamination reads had significantly higher numbers of contigs, degree of contaminations, and lower ANI, but N50, completeness, length of largest contigs, and genome length did not differ significantly (Fig. 5A–D, and Supplementary Fig. S3A–C). Our results showed that it was difficult for CheckM to identify low rates of sequence contamination. To evaluate the impact of insert size on genome assembly, 200-600bp insert sizes were applied for sequence simulation. There was no significant difference in assembly assessment parameters, completeness, contamination, and ANI between assemblies for different insert sizes in reads simulation (Fig. 5E,F, and Supplementary Fig. S3D,E).

## Discussion

The cPAS-based BGI DNBSEQ sequencer has been commonly used and shown to perform well in eukaryotic genome sequencing^8^ and metagenomic sequencing^19^. Considering the increasing demand for cultivated bacterial genome sequencing, the DNBSEQ platform seems as an excellent candidate for bacterial genome research. To evaluate the performance of the DNBSEQ platform, we compared genomes assembled from BGISEQ-500 sequencing data and Illumina HiSeq 2000 sequencing data of 76 strains by detecting and comparing the completeness, contamination, genome assembly quality, 16S rRNA genes, mutations, and metagenomic read mapping. The values of most assembly parameters of genomes from the two sequencing strategies were very close. HiSeq 2000 has a little better performance in relation to the length of the largest contigs and N50, and the numbers of contigs and N bases per 100Kb. The completeness of BGISEQ-500 genome assemblies was higher, with similar results obtained for genome assemblies of high and low GC content. We noted that the numbers of N bases per 100Kb were lower in BGISEQ-500 genomes of high GC content. Although smaller insert sizes may have a higher priority in DNB sequencing, the results showed that insert size had no significant impact on genome assembly.

The 16S rRNA gene is a frequently used marker gene in the taxonomy analyses of bacteria. 16S rRNA genes from BGISEQ-500 genomes and HiSeq 2000 genomes were extremely close in the sequence similarity and there was no significant difference in gene length. In addition, the comparison with genome distance algorithms of ANI, AAI, Mash, and Tetra supported the high similarity between BGISEQ-500 assemblies and HiSeq 2000 assemblies. Furthermore, we calculated the numbers of SNV and functional genes, and the follow-up comparison showed that the use of the two platforms had no significant impact on the detection of mutation at the single nucleotide level and in the functional annotation of bacterial genomes. The BGISEQ-500 platform appeared to have higher efficiency in deletion calling, but lower in insertion calling. Culture-independent metagenomic studies have used cultivated bacterial genomes and metagenome-assembled genomes (MAGs) to build customized databases for metagenome classification and calculation of bacterial relative abundance by metagenomic reads mapping^23–26^. To assess the metagenomic read classification performance, customized genomic databases of BGISEQ-500 genome assemblies and HiSeq 2000 genome assemblies were built and mapped against metagenomic sequencing data by Kraken2 and Bracken. Comparison of relative abundances and beta-diversity analyses showed that the distribution of genome assemblies from the two platforms was extremely consistent.

The Illumina platforms produce accurate sequencing data rapidly and have been widely used in genome sequencing of eukaryotes and prokaryotes, and metagenome sequencing. The DNBSEQ sequencer perform better in the comparison of sequencing error rates^8^. Compared with Illumina platforms, the DNBSEQ platform was shown to be applicable for metagenomic studies providing high accuracy and technical reproducibility^19^. In this work, we compared the assemblies of BGISEQ-500 sequencing reads and HiSeq 2000 sequencing reads by genome assembly assessment, sequence similarity analysis of 16S rRNA genes and genomes, mutation detection, and metagenomic reads mapping demonstrating excellent performance and applicability of the BGISEQ-500 platform for bacteria genome sequencing, as also demonstrated in our recent work^21^. Besides BGISEQ-500 and Illumina HiSeq 2000, more upgraded sequencers have been produced, including DNBSEQ-T20, Illumina NovaSeq and NextSeq 1000/2000, more comparison (cost, index hopping) should be conducted on these newer platforms.

## Methods

### Genome sequencing, assembling, and quality assessment

Whole-genome sequencing was performed using BGISEQ-500 and HiSeq 2000 as described previously^19^. SOAPdenovo (v2.04)^29^ was used for de novo assembly of sequencing reads. CheckM (v1.0.13)^30^ was used to evaluate the completeness and contamination of genomes. QUAST (v5.0.2)^31^ was used to assess the quality of genome assemblies and conduct paired comparison with parameters ‘-f’ and ‘-r’. Unconstrained principal coordinates analysis (PCoA) based on Jaccard dissimilarity of all features in the result of QUAST was conducted using the R function ‘vegdist’ and ‘pcoa’.

### Taxonomy annotation and 16S rRNA gene prediction

GTDB-Tk (v204, database release 214, ‘classify_wf’ function and default parameters)^32^ was used to perform taxonomic annotation of each genome. Reference genomes were downloaded from the NCBI Genome database by searching the species name identified by GTDB-Tk. 16S ribosomal RNA coding regions of genome assemblies from BGISEQ-500, HiSeq 2000, and NBCI-downloaded references were predicted using Barrnap (https://github.com/tseemann/barrnap). We used an in-house script to extract 16S rRNA genes and calculate gene length. BLAST was used to determine the sequence identity of 16S rRNA genes between BGISEQ-500 assemblies and HiSeq 2000 assemblies.

### Calculation of ANI, AAI, tetra correlation, and mash distance

Pairwise comparisons for genomes of the same strain from BGISEQ-500 and HiSeq 2000 sequencing platforms were performed by the calculation of pairwise ANI, AAI, Tetra correlation, and Mash distance. FastANI (v1.32)^33^, CompareM (v0.1.2, https://github.com/dparks1134/CompareM), pyani (v0.2.11, https://github.com/widdowquinn/pyani) and Mash (v2.3)^34^ were used to calculate ANI, AAI, Tetra correlation, and Mash distance.

### Identification of SNV and InDel and genome collinearity

Whole-genome alignments of genome assemblies from the same strain were created with the Parsnp (v1.5.6)^35^ using NCBI downloaded genomes belonging to the same species as references and MAFFT as an alignment program. Harvesttools (v1.2)^35^ was subsequently used to extract SNV. MUMmer (v3.23)^36^ toolkit was additionally used for reference mapping (nucmer), filtering (delta-filter), and SNV/InDel detection (show-snps). We used an in-house script to calculate the numbers of SNV and InDel.

### Genome collinearity, genome annotation, and BUSCO assessment

Analysis of genomic collinearity among genome assemblies and references was conducted by the MCScanX software. Genomic comparison was visualized with proksee (https://proksee.ca/). Prokka (v1.13.4)^37^ was used to predict genes and generate gene annotation, including COGs (Clusters of Orthologous Genes), enzymes, gene names, and RNA. BUSCO (v5.1.2, Benchmarking Universal Single-Copy Orthologs)^38^ was used to assess genome completeness and generate the numbers of ‘Complete’ BUSCOs, ‘Complete and single-copy’ BUSCOs, ‘Complete and duplicated’ BUSCOs, ‘Fragmented’ BUSCOs, and ‘Missing’ BUSCOs with bacteria_odb10 as the only reference. In-house R/shell scripts were used to summarize the outputs and compare BGISEQ-500 and HiSeq 2000 regarding the numbers of annotated genes or BUSCOs.

### Distribution of genome assemblies from BGISEQ-500 and HiSeq 2000 in a metagenome cohort

Human gut metagenome sequencing data of a Chinese cohort (a part of 4D-SZ^39^) were downloaded from the CNGB Sequence Archive (CNSA)^27^ (https://db.cngb.org/cnsa/) of China National GeneBank DataBase (CNGBdb)^28^ under the accession code CNP0000426. The 152 assemblies of 76 strains were built as a BGISEQ-500 custom genome database and a HiSeq 2000 custom genome database by Kraken2^40^ and Bracken^41^. In addition, Kraken2 and Bracken were used to map the reads of the Chinese metagenome cohort to the two databases. The median and mean of the relative abundances of the BGISEQ-500 and HiSeq 2000 assemblies in the Chinese cohort were calculated, and the correlations between the medians and means of paired assemblies were analyzed based on Spearman’s rank correlation coefficient. R function vegdist (Bray–Curtis dissimilarity) and R function pcoa were used to perform PCoA, and the R function envfit was used to test the correlation of platforms and the PCoA coordinates.

### Sequencing reads simulation

Dwgsim was used to simulate sequencing data with parameters ‘-1 100 -2 100 -r 0 -R 0 -X 0 -e 0 -E 0 -N 30000’. NCBI-downloaded genomes were used as the template. Three million reads were produced by dwgsim for each genome as clean reads. To produce contamination in sequencing reads, (1) all reference genomes were pooled together, (2) simulating 0%*3M, 0.5%*3 M, 1%*3 M, 2%*3 M, 4%*3 M, and 7%*3 M reads from pooled genomes as the contamination, (3) mixing clean reads with contamination reads. In addition, insert sizes of 200 bp, 300 bp, 400 bp, 500 bp, and 600 bp were used for reads simulation. Genome completeness and contamination were calculated with CheckM^30^. FastANI was also used to calculate ANI between assemblies and reference genomes. Wilcoxon rank test and ANOVA were used to conduct statistical analysis.

### Supplementary Information

Supplementary Figures.

Supplementary Legends.

Supplementary Table 1.

Supplementary Table 2.

Supplementary Table 3.

Supplementary Table 4.

Supplementary Table 5.

Supplementary Table 6.

Supplementary Table 7.

## Data Availability

The 76 bacterial strains in this article have been deposited in China National GeneBank (CNGB), a non-profit, public-service-oriented organization in China. The data that support the findings of this study have been deposited into the CNGB Sequence Archive (CNSA)^27^ of China National GeneBank DataBase (CNGBdb)^28^. The 76 Illumina HiSeq 2000 assemblies can be downloaded from CNSA (https://db.cngb.org/search/project/CNP0000126/, https://db.cngb.org/search/project/CNP0001833/). The 76 BGISEQ-500 assemblies are publicly available from https://db.cngb.org/search/project/CNP0003311/. The Chinese gut metagenome sequencing data can be found and accessed through https://db.cngb.org/search/project/CNP0000426/. The scripts of SNV and InDel calling, and reads simulation are publicly available through Github (https://github.com/hutongyuan/BGISEQ-500_VS_HiSeq-2000).
