# Supplementary material for: Comparison of the DNBSEQ platform and Illumina HiSeq 2000 for bacterial genome assembly
Source: Sci Rep. 2024 Jan 14;14:1292. doi: 10.1038/s41598-024-51725-0 (PMC10788345; doi:10.1038/s41598-024-51725-0)
Supplement: Supplementary file 1 — Supplementary Figures. [file 41598_2024_51725_MOESM1_ESM.docx]

**Supplementary information:**

**Comparison of BGISEQ-500 and Illumina HiSeq 2000 for bacterial genome assembly**

Tongyuan Hu^1,2^, Jianwei Chen^1,3^, Xiaoqian Lin^1,4^, Wenxin He^1^, Hewei Liang^1,2^, Mengmeng Wang^1^, Wenxi Li^1,4^, Zhinan Wu^1^, Mo Han^1,5^, Xin Jin^1^, Karsten Kristiansen^1,3,5^, Liang Xiao^1,3,6^, Yuanqiang Zou^1,3,5,6*^

^1^ BGI Research, Shenzhen 518083, China

^2^ BGI Research, Wuhan, 430074, China

^3^ Lars Bolund Institute of Regenerative Medicine Qingdao-Europe Advanced Institute for LifeSciences, BGI Research, Qingdao 266555, China

^4^ School of Bioscience and Biotechnology, South China University of Technology, Guangzhou 510006, China

^5^ Laboratory of Genomics and Molecular Biomedicine, Department of Biology, University of Copenhagen, Universitetsparken 13, 2100 Copenhagen, Denmark

^6^ Shenzhen Engineering Laboratory of Detection and Intervention of Human Intestinal Microbiome, BGI Research, Shenzhen 518083, China

^*^corresponding [zouyuanqiang@genomics.cn](mailto:zouyuanqiang@genomics.cn)

**Supplementary Figures**


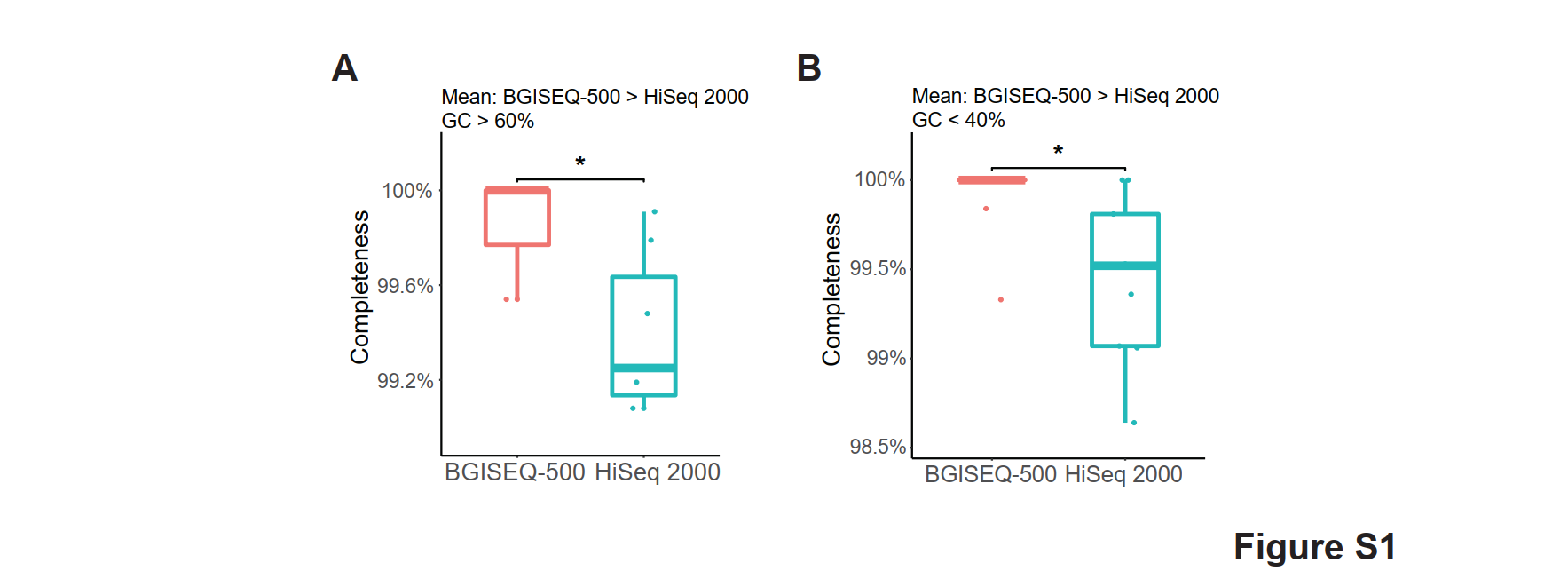


**Supplementary Figure S1. Completeness of high-GC (a) and low-GC (b) genome assemblies.** High-GC assembly, GC content > 60%. Low-GC assembly, GC content < 40%. Mean, mean completeness of BGISEQ-500 assemblies or HiSeq 2000 assemblies.

**
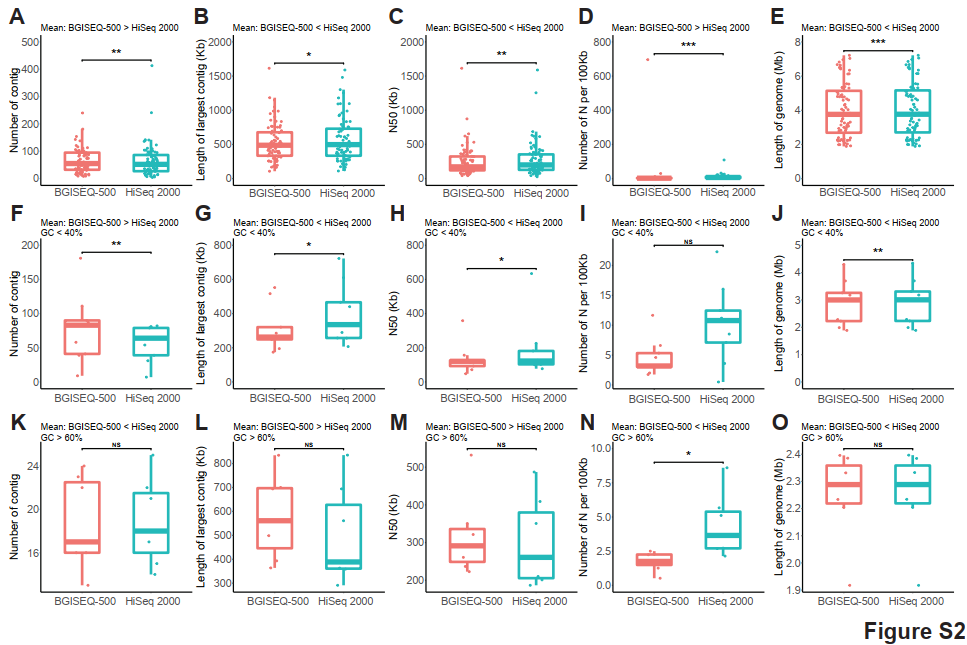
**

**Supplementary Figure S2. Quality assessment of genome assemblies.** (a, f, k) Number of contig. (b, g, l) length of largest contig. (c, h, m) N50. (d, i, n) Number of N per 100Kb. (e, j, o) Genome length. (a-e) All BGISEQ-500 assemblies and HiSeq 2000 assemblies. (f-j) Low-GC BGISEQ-500 assemblies and low-GC HiSeq 2000 assemblies. (k-o) High-GC BGISEQ-500 assemblies and high-GC HiSeq 2000 assemblies.

**
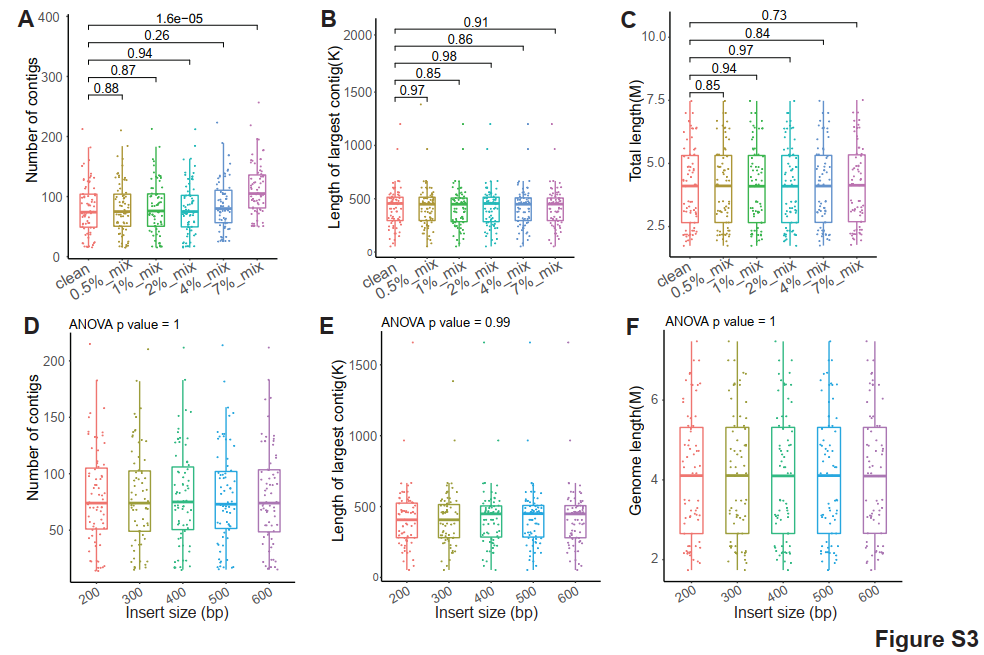
**

**Supplementary Figure S3. The impact of index hopping (a-c) and insert size (d-f) on assembly.** (a, d) Number of contig. (b, e) Length of largest contig. (c, f) Genome length. (a-c) The quality assessment and comparison of genome assemblies generated from simulated reads mixed with 0-7% contamination. (d-f) The quality assessment and comparison of genome assemblies generated from simulated reads with insert sizes 200-600bp.
