# Supplementary material for: Comparison of the DNBSEQ platform and Illumina HiSeq 2000 for bacterial genome assembly
Source: Sci Rep. 2024 Jan 14;14:1292. doi: 10.1038/s41598-024-51725-0 (PMC10788345; doi:10.1038/s41598-024-51725-0)
Supplement: Supplementary file 2 — Supplementary Legends. [file 41598_2024_51725_MOESM2_ESM.docx]

**Description of Additional Supplementary Files**

**File Name:** Supplementary Table S1

**Description:** Taxonomic and source information of the 76 bacterial strains

**File Name:** Supplementary Table S2

**Description:** Detailed information of quality assessment of genome assemblies

**File Name:** Supplementary Table S3

**Description:** Information of downloaded genome references

**File Name:** Supplementary Table S4

**Description:** Percentage of collinear genes

**File Name:** Supplementary Table S5

**Description:** Summary and comparison of genomic functional annotation by Prokka

**File Name:** Supplementary Table S6

**Description:** Detailed information from the BUSCO assessment tool

**File Name:** Supplementary Table S7

**Description:** Relative abundance of the 76 paired genome assemblies in the Chinese metagenome cohort
